# Distribution of Rhodium in Mice Submitted to Treatment With the Adduct of Rhodium Propionate and Sodium Isonicotinate

**DOI:** 10.1155/MBD.1997.39

**Published:** 1997

**Authors:** Aparecido Ribeiro de Souza, Renato Najjar, Elizabeth de Oliveira, Szulim Ber Zyngier

**Affiliations:** 1 Instituto de Química USP C.P. 26.077 São Paulo SP 05599-970 Brazil; 2 Instituto de Ciências Biomédicas USP C.P. 66.208 São Paulo SP 05383-970 Brazil

## Abstract

The distribution of rhodium in Balb/c mice following intraperitoneal (ip) administration of a solution of adduct of rhodium propionate and sodium isonicotinate has been investigated. The metal concentration was determined in blood and in the following organ tissues: brain, heart, lung, liver, spleen, kidney, testes, and uterus/ovary, and the rhodium concentration was obtained by Inductively Coupled Argon Atomic Emission Spectroscopy (ICP-AES). The metal was detected in all organ tissues examined, mainly in spleen, liver, uterus/ovary and heart. Nine days after the injection, traces of rhodium were found in the liver and kidneys and, twenty days after the injection, only in the liver.


### DISTRIBUTION OF RHODIUM IN MICE SUBMITTED TO TREATMENT WITH THE ADDUCT OF RHODIUM PROPIONATE AND SODIUM ISONICOTINATE

Aparecido Ribeiro de Souza*[1], Renato Najjar[1], Elizabeth de Oliveira[1] and Szulim Ber Zyngier[2]

[1] Instituto de Química, USP, C.P. 26.077, 05599-970 São Paulo - SP, Brazil

[2] Instituto de Ciências Biomédicas, USP, C.P. 66.208, 05383-970 São Paulo - SP, Brazil

**Abstract**
The distribution of rhodium in Balb/c mice following intraperitoneal (ip) administration of a solution of adduct of rhodium propionate and sodium isonicotinate has been investigated. The metal concentration was determined in blood and in the following organ tissues: brain, heart, lung, liver, spleen, kidney, testes, and uterus/ovary, and the rhodium concentration was obtained by Inductively Coupled Argon Atomic Emission Spectroscopy (ICP-AES). The metal was detected in all organ tissues examined, mainly in spleen, liver, uterus/ovary and heart. Nine days after the injection, traces of rhodium were found in the liver and kidneys and, twenty days after the injection, only in the liver.

### INTRODUCTION

Since the introduction of cisplatin into clinical practice against human malignant neoplasms [1], several metal complexes, including rhodium carboxylates, have been tested for antitumor activity [2-4]. The rhodium(II) carboxylates have the common tetrabridged acetate structure with a short Rh-Rh bond (Figure 1), whose axial positions can be readily occupied by Lewis bases [5,6].

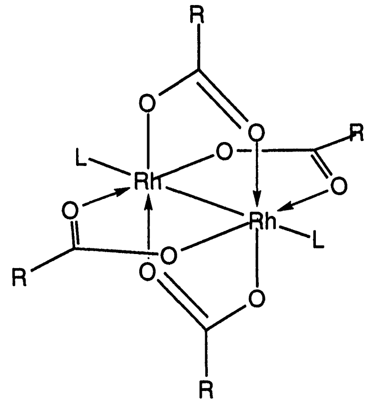

Figure 1. General molecular structure of the rhodium(II) carboxylates with two labile positions (L).

The pharmacological evaluations of rhodium carboxylates (rhodium acetate, propionate and butyrate), have shown statistically significant activity against Ehrlich ascites carcinoma, melanoma B16, leukemia P388 and leukemia L1210 [2,7,8]. The activity was found to be in the order: acetate < propionate < butyrate; rhodium(II) propionate having a more interesting therapeutic index. However, the researches involving these complexes have diminished, in part because of the demonstrated acute toxicity [9,10] as well as the evident difficulty of aqueous solubilization of most of the compounds synthesized.
Recently, we reported a strategy for turning these compounds more water-soluble, by forming adducts with isonicotinic acid [11], that allows more appropriate experimental investigation of the activity of rhodium carboxylates on tumor cells.
As an extension of these studies and as part of the pharmacological evaluation of rhodium(II) carboxylates, we have studied the distribution of rhodium in mice Balb/c following the i.p. administration of the adduct of rhodium(II) propionate and sodium isonicotinate.

### MATERIALS AND METHODS

*Chemicals*

The adduct of rhodium(II) propionate and isonicotinic acid was prepared by the method previously described [11]. The compound was dissolved in sodium hydrogen carbonate solution immediately prior to administration.

*Tissue distribution studies*

Male and female Balb/c mice (15-20 g) were used throughout this study. The animals received doses of 30 mg/kg (3.5 times DL10 [11]) ip, and were killed serially (3 males and 3 females per time point) by cervical dislocation. Blood samples were collected immediately by cardiac puncture. Whole organs or representative tissue samples were removed and weighed, when necessary.

*Tissue analysis*

The samples were analyzed for their rhodium content using an Inductively Coupled Argon Atomic Emission Spectrophotometer (model SpectraFlame Modula), using the line emission Rh 343.489 nm. Tissue (up to 50 mg) and blood (up to 0.10 mL) samples were digested in concentrated nitric acid (2 mL) for 24 h. After tissue digestion, an attempt to clear the solution was made, adding 30% hydrogen peroxide (200 µL), followed by a gentle heating (30 min). The resultant solution was completed to 5 mL with distilled water. The method permits estimation of total rhodium metal in biological samples and no attempt was made to identify the state of the metal ion present. A calibration curve was obtained using standard solutions in the range of 1.025-1025 µg/mL. This method has a detection limit up to 15.2 µg/L.

*Pharmacokinetic analysis*

The pattern of compound from the blood or tissue (liver and kidney) is described by a one-compartment model and the constants determined are shown in Table 2.

### RESULTS

The results, presented in Table 1, were obtained by averaging six values obtained for organs of three males and three females, except those for testes and uterus/ovary that were obtained from three values. Standard deviations were less than 20% of the means. The tissue distribution of total rhodium is shown in Table 1.

TABLE 1. Tissue distribution of rhodium in Balb/c mice after intraperitonial administration.

| Tissue | Rhodium concentration[a] | | | | | | |
|---|---|---|---|---|---|---|---|
| | 1 h | 8 h | 24 h | 48 h | 6 days | 9 days | 20 days |
| Blood | 40±4 | 42±4 | 23±2 | 15.0±0.5 | 2.4±0.2 | ND | ND |
| Brain | 16±3 | 9.9±0.4 | 15±1 | 9.7±0.3 | 0.20±0.02 | ND | ND |
| Heart | 46±3 | 51±2 | 46±2 | 50±2 | 11.3±0.8 | ND | ND |
| Lung | 31±1 | 32±3 | 37±2 | 32±3 | 3.1±0.4 | ND | ND |
| Liver | 38±5 | 43±4 | 57±5 | 51±2 | 23±3 | 16±2 | 6.5±0.4 |
| Spleen | 79±8 | 96±8 | 100±6 | 96±5 | 9.6±0.8 | ND | ND |
| Kidney | 25±1 | 29±2 | 31±1 | 28±1 | 8±1 | 4±2 | ND |
| Testes | 32±5 | 26±5 | 40±7 | 28.8±0.6 | 1.5±0.5 | ND | ND |
| Uterus/Ovary | 66±6 | 71±7 | 58±6 | 41±3 | 7±1 | ND | ND |

[a] The mean (N =6) rhodium concentrations are expressed in micrograms per milliliter (µg/mL) for blood and micrograms per gram of wet weight (µg/g) for the tissues. Mean ± SD; ND: not detectable

Spleen, liver and uterus/ovary are the tissues that showed the highest concentrations of rhodium. The rhodium persisted in the majority of the tissues for at least six days, disappearing after this period. In liver, the metal remained more than twenty days. Table 1 indicates that the maximal rhodium concentration in the tissues was reached at 24-48 h, declining after this period.

The blood, liver and kidney rhodium time data were fitted to a one-compartment model and the results are shown in Table 2. The tissue half-life of rhodium is about 2-fold greater for liver than for kidney.

### DISCUSSION

The interest in the potential antitumor activity of rhodium(II) carboxylates has gained new strength in recent years. Several biological studies have been performed in vitro using tumor cell lines [11,12] and in vivo with mice bearing tumors [10], searching for more effective compounds. However, there is surprisingly little information concerning the distribution of these rhodiumcomplexes in the mice organism. Giraldietal. [13] found that, following administration of bis(cycloocta-1,5-diene)-μ,μ'-dichlorodirhodium(I) (40 mg/kg) and bis(l,5-hexadiene)-μ,μ'-dichlorodirhodium(I) (20 mg/kg) complexes, rhodium accumulates in liver and spleen after 4 hours.

TABLE 2. Constants and half-lives for rhodium following a one-compartment pharmacokinetic analysis.

| Tissue | Pharmacokinetic Parameters | |
|---|---|---|
| | $k_{el}$ ($h^{-1}$)[a] | $t_{1/2}$(h)[b] |
| Blood | 0.0206 | 33.6 |
| Liver | 0.0046 | 149 |
| Kidney | 0.0112 | 62. 0 |

[a] Elimination constant [b]Terminal elimination half-life

The results shown in Table 1 indicate that, after ip injection (30 mg/kg), the compound was rapidly absorbed and reached maximum blood concentration, c.a. 42,ug/mL, within eight hours. In 24-48 hours, all tissues reached maximal rhodium concentrations, with spleen attaining the higher metal content. After six days, small amounts of rhodium were found only in liver and kidney. After twenty days, the rhodium content falls to non-detectable levels for all the tissues except the liver.
Comparing our results (Table 2) with those published for cisplatin and carboplatin in mice [14], we note that the blood half-life time ($t_{1/2}$) of rhodium (33.6 h) is slightly shorter than that described for platinum in cisplatin (36.5 h) or carboplatin (49.5 h). Likewise, the t.,2 of rhodium in liver (149 h) is similar to that for platinum in cisplatin (133 h) and carboplatin (147 h). However, the t.,2 of rhodium in kidney (62.0 h) is shorter that for platinum in cisplatin (119 h) and carboplatin (94.9 h).

**Acknowledgments**
The authors are grateful to the Conselho Nacional de Desenvolvimento Cientifico e Tecnologico (CNPq) and the Fundacao de Amparo a Pesquisa do Estado de Sao Paulo (FAPESP) for financial support and a post-doctoral fellowship (ARS, FAPESP).

**REFERENCES**

1. Cleare, M.J. *Inorg. Perspect. Biol. Med.*. 1977, **1**, 19.
2. Bear, J.L., Howard, R.A. and Dennis, A.D. *Curr. Chemother. Proc. Int. Congr. Chemother 10th*, 1977, 1978, **2**, 1321.
3. Hall, L.M., Speer, R.J. and Ridgway, H.J. *J Clin. Hematol. Oncol.* 1980, **10**, 25.
4. Zyngier, S.B., Kimura, E. and Najjar, R. Braz. *J. Med. Biol. Res.* 1989, **22**, 397.
5. Bear, J.L., Howard, R.A. and Korn, J.E. *Inorg. Chim. Acta.* 1979, **32**, 123.
6. Koh, Y.-B. and Christoph, G.G. *Inorg. Chem.* 1979, **18**, 1122.
7. Erck, A., Rainen, L., Whileyman, J., Chang, I.M., Kimball, A.P. and Bear, J.L. *Proc. Soc. Exp. Biol. Med.* 1974, **145**, 1278.
8. Howard, R.A., Kimball, A.P. and Bear, J.L. *Cancer Res.* 1979, **39**, 2568.
9. Howard, R.A., Sherwood, E., Erck, A., Kimball, A.P. and Bear, J.L. *J Med. Chem.* 1977, **20**, 943.
10. Nothenberg, M.S., Zyngier, S.B., Giesbrecht, A.M., Gambaerdella, M.T.P., Santos, R.H.A., Kimura, E. and Najjar, R. *J. Braz. Chem. Soc.* 1994, **5**, 23.
11. Souza, A.R. de, Najjar, R., Glikmanas, S., and Zyngier, S.B. J: Inorg. Biochem. 1996, 64, 1.
12. Pruchnik, F. and Dus, D. *J. Inorg. Biochem.* 1996, **61**, 55.
13. Giraldi, T., Sava, G., Mestroni, G., Zassinovich, G. and Stolfa, D. *Chem. Biol. Interact.* 1978, **22**, 231.
14. Siddik, Z.H., Jones, M., Boxall, F.E. and Harrap, K.R. *Cancer Chemother. Pharmacol.* 1988, **21**, 19.

**Received: December 5, 1996 - Accepted: January 7, 1997 - Received in revised camera-ready format: February 17, 1997**

